# Applications of a Rapid and Sensitive Dengue DUO Rapid Immunochromatographic Test Kit as a Diagnostic Strategy during a Dengue Type 2 Epidemic in an Urban City

**DOI:** 10.1371/journal.pone.0158437

**Published:** 2016-07-14

**Authors:** Hsin-I Shih, Hsiang-Chin Hsu, Chi-Jung Wu, Chih-Hao Lin, Chia-Ming Chang, Yi-Fang Tu, Chih-Chia Hsieh, Chih-Hsien Chi, Tzu-Ching Sung

**Affiliations:** 1 Department of Emergency Medicine, National Cheng Kung University Hospital, National Cheng Kung University Tainan, Taiwan; 2 Department of Emergency Medicine, National Cheng Kung University Hospital, College of Medicine, National Cheng Kung University, Tainan, Taiwan; 3 National Institute of Infectious Diseases and Vaccinology, National Health Research Institutes, Tainan, Taiwan; 4 Department of Public Health, National Cheng Kung University Hospital, College of Medicine, National Cheng Kung University, Tainan, Taiwan; 5 Division of Geriatrics, Department of Internal Medicine, National Cheng Kung University Hospital, Tainan, Taiwan; 6 Division of Geriatrics, Department of Internal Medicine, National Cheng Kung University Hospital, College of Medicine, National Cheng Kung University, Tainan, Taiwan; 7 Department of Pediatrics, National Cheng Kung University Hospital, College of Medicine, National Cheng Kung University, Tainan, Taiwan; 8 Department of Health Care Management, University of Kang Ning, Tainan, Taiwan; 9 National Mosquito-Borne Diseases Control Research Center, National Health Research Institutes, Tainan, Taiwan; University of Malaya, MALAYSIA

## Abstract

Dengue infection is a major health problem in tropical and subtropical countries. A prospective observational study in a university-affiliated hospital was conducted between August 2015 and September 2015. Patients who visited the emergency department (ED) with a presentation of any symptoms of dengue were eligible for the dengue non-structural protein 1 (NS1), IgM/IgG rapid immunochromatographic tests and real-time polymerase chain reaction (RT-PCR) to evaluate the performance of the rapid tests. Considering the RT-PCR as the gold standard for the dengue diagnosis, the ideal primary results of sensitivity (80–100%), specificity (60–84%), positive predicted value(75%-95%), and negative predicted value (70–100%) suggested that the NS1-based test with or without a combination of IgM and IgG tests have good diagnostic performances in detecting dengue infections, even in the afebrile or elderly populations.

## Introduction

Dengue infection caused by four main types of dengue virus (DENV) is a major health problem in tropical and subtropical countries. Each year, more than 250,000 cases of dengue hemorrhagic fever (DHF) and dengue shock syndrome (DSS) are reported from an estimated 50 million dengue infections [1–3]. Dengue is transmitted between people by the mosquitoes *Aedes aegypti* and *Aedes albopictus*, which are found throughout the world. Clinical presentations of dengue diseases range from asymptomatic or self-limiting dengue fever (DF) to severe dengue characterized by plasma leakage (DHF, grades 1 and 2) that can lead to a life-threatening syndrome (DSS, grades 3 and 4) and severe bleeding and/or severe organ impairment [1]. Severe dengue-related fatal cases usually present with DSS, and the mortality of DSS is reportedly 50 times higher than that of dengue patients without DSS [4].

Taiwan is located in the tropical and subtropical area of the West Pacific Rim. Each year, the annual dengue epidemic reported in Taiwan with imported and domestic cases range from 3,000 to 10,000. Several large-scale dengue outbreaks occurred in the past two decades in southern Taiwan, mostly in Kaohsiung. A substantial number of severe dengue type 2 cases was identified during the large-scale epidemic in 2002 [5]. Another large-scale outbreak of dengue with 8,636 reported cases was regarded to be associated with typhoons and sewage system malfunctions after a pipeline explosion in 2014 [5–7].

In 2015, a recent large-scale dengue outbreak severely affected another major city in southern Taiwan, Tainan, which is a city with a population of 1.8 million and with 780,000 people living in the metropolitan areas. Historically, the number of annual dengue cases in Tainan was approximately 1,000. During this large outbreak, approximately 20,000 patients and 130 fatal patients were observed from August to October in 2015 [8].

Accurate and timely identification of the emerging infectious disease would lead to early initiation of appropriate treatment and further prevention and management plans. Emergency departments (EDs) are important locations for the diagnosis and management of infectious diseases [9, 10]. A large census of patients flooded the health care facilities during epidemics of infectious diseases. The overcrowded care units result in a heavy workload for the health care professionals and a deteriorated quality of care that is provided in the health care facilities [11, 12]. Therefore, proposing a rapid and accurate diagnosis tool could improve the care delivered in overcrowded health care facilities during an epidemic of infectious diseases. The Centers for Disease Control, Taiwan, decided to introduce the rapid test, Dengue non-structural protein 1 (NS1), IgM, IgG, as the major diagnostic tool in major health facilities. Patients who visited the EDs with presentation of any symptoms of dengue, such as fever, malaise and who lived in the endemic areas, were eligible to receive the dengue rapid tests and a real-time polymerase chain reaction (RT-PCR). This study aimed to examine the performance and the accuracy of the dengue rapid test as a rapid diagnostic tool in an endemic area having an abruptly increased census in an ED. The overcrowding in the ED occurred during an initial dengue outbreak in an urban city in southern Taiwan.

## Materials and Methods

### Study Design and Setting

We conducted an observational study from August 2015 to September 2015 in a tertiary teaching hospital in southern Taiwan outfitted with 1000 beds, 100 of which were intensive care unit (ICU) beds. The modified Canadian Triage & Acuity Scale (CTAS) was applied as the triage tool (Taiwan triage and acuity scale, TTAS) [13]. The annual number of patients treated in the study ED was approximately 89,700 in 2014. The mean length of stay (LOS) in the ED and the LOS for patients who left following treatment in the ED were 6.89 hours and 4.8 hours, respectively, in 2014.

### Sample Collection

Patients’ data were collected from electronic medical records. Totally, there were 1607 patients meting the dengue survey criteria enrolled in the study. The survey criteria included patients who presented any of the symptoms such as fever, generalized malaise, post-orbital headache, and lived in the epidemic neighborhood. The obtained information included triage level, initial vital signs, length of stay, underlying diseases, diagnosis, time of admission to and departure time from the ED, and patient disposition. The International Classification of Diseases, Ninth Revision, Clinical Modification (ICD-9-CM) ICD-9 codes used for the chronic diseases were as follows: The International Classification of Diseases, Ninth Revision, Clinical Modification (ICD-9-CM) ICD-9 codes used for the chronic diseases were as follows: diabetes mellitus (DM)-250, chronic obstructive pulmonary disease (COPD) (chronic bronchitis-490-491; emphysema-492; bronchiectasis-494; chronic airway obstruction 496), malignant neoplasms-140-239, chronic kidney disease (CKD)-585, 5839, and chronic liver disease and cirrhosis-571.

### Ethics Statement

All provisions of the study were in accordance with the Helsinki Declaration. Both of the study protocol and data have been approved by the Institutional Review Board (IRB), National Cheng Kung University Hospital (A-ER-104-223). To protect personal privacy, the electronic databases were decoded for research. Patient information was collected and made anonymous and patients were de-identified prior to analysis, thereby the requirement for informed consent was waived by the IRB.

### Dengue Diagnosis and Diagnostic Tools

Five to ten ml blood samples were drawn to test complete blood counts (CBC), renal and liver functions, and dengue. During the study period, 5–10 μl of serum was drawn for detection of nonstructural protein 1 (NS1) and the IgM/IgG using the SD BIOLINE Dengue DUO® rapid immunochromatographic test kit (Standard Diagnostics, Inc., Kyonggi-do, Korea) [14]. A real-time polymerase chain reaction (RT-PCR) using a Lightmix® kit which detects Dengue virus types 1, 2, 3 and 4 (TIB MOLBIOL GmbH, Berlin, Germany) were conducted to verify the diagnosis (based on the manufacturer’s instruction). Either NS1, IgM/IgG or RT-PCR tests were ordered to diagnose dengue, based on the clinical judgement of the physicians. Positive RT-PCR results were regarded as the gold standard for the diagnosis of dengue.

### Statistical Analysis

Descriptive statistics and cross tabulations were calculated for the frequency distribution of cases examined using the NS1, IgM/IgG, and RT-PCR screening tests. We used Cochran-Armitage trend test to quantify trends in the binomial proportions across the levels of a single screening. The Cochran-Armitage trend test is appropriately applied when the screen variable has two levels, i.e., negative and positive, and the other variable (fever days) is ordinal. Regression methods were used to analyze the best fit for a log-transformed viremia titer by body temperature, which helped with forecasting, modeling, and calculating R squared values (the coefficient of determination), the 95% confidence band and the lower and upper 95% prediction limits. We also used Spearman Rank and Pearson correlation coefficients to measure the strength and direction of a linear relation between the dengue viremia titer and body temperature on a scatterplot. SAS software version 9.4 (SAS, Inc., Cary, North Carolina, USA) was applied to data entry, processing and statistical analysis. Statistical significance was achieved when p was <0.05.

## Results

### Demographic Data

For the 1607 patients who met the dengue survey criteria and were enrolled in the study, the demographic characteristics presented in the ED are summarized in Table 1. Of the 1607 patients, there were 1295 patients examined using the rapid dengue NS1 antigen test, 1266 patients examined using the IgM/IgG tests, and 534 cases examined using the RT-PCR test. Of the 1295 patients receiving the NS1 test, there were more females than males (50.2% vs. 49.8%, respectively). The majority of the cases were adults (0–17 years, 7.4%; 18 to 64 years, 63.9%; 65 years and over, 28.7%).

**Table 1:** Clinical characteristics among 1607 enrolled cases who tested for NS1, IgM/IgG, RT-PCR.

Characteristics	NS1	IgM/IgG	RT-PCR
	(n = 1295)	(n = 1266)	(n = 534)
	No.(%)	No.(%)	No.(%)
**Sex**			
Female	650(50.2)	636(50.2)	267(50.0)
Male	645(49.8)	630(49.8)	267(50.0)
**Age (years)**			
Mean ± SD	49.27 ± 21.78	49.37 ± 21.64	50.16 ± 20.78
0–17 years	96(7.4)	89(7.03)	17(3.18)
18–64	828(63.9)	816(64.4)	362(67.8)
≥ 65	371(28.7)	361(28.5)	155(29.0)
**Temperature (°C)**			
Mean ± SD	38.33 ± 0.96	38.31 ± 0.96	38.19 ± 0.96
**Respiration rate (per minute)**			
Mean ± SD	19.85 ± 3.88	19.84 ± 3.91	19.67 ± 1.58
**Diastolic Blood Pressure (mmHg)**			
Mean ± SD	81.87 ± 15.20	81.97 ± 15.23	81.69 ± 16.88
**Systolic Blood Pressure (mmHg)**			
Mean ± SD	134.01 ± 21.43	134.01 ± 21.42	133.61 ± 21.43
**Mean Artery Pressure (mmHg)**			
Mean ± SD	99.25 ± 15.67	99.31 ±15.68	99.00 ± 16.45
Pulse rate (/minute)			
Mean ± SD	100.89 ± 19.61	100.64 ± 19.63	98.68 ± 18.80
**Length of Stay (hours)**			
Mean ± SD (overall)	7.55 ± 11.13	7.31 ± 10.57	8.94 ± 12.28
AAD	16.59 ± 17.01	15.72 ± 16.72	26.58 ± 15.80
Admission	21.89 ± 17.14	21.13 ± 17.15	22.64 ± 18.49
Expired	7.18 ± NA	7.18 ± NA	NA
MBD	5.61 ± 8.46	5.47 ± 7.82	6.84 ± 9.73
Transfer	16.57 ± 13.94	16.57 ± 13.94	15.77 ± 13.96
**Deposition**			
AAD	27(2.1)	26(2.1)	7(1.3)
Admission	119(9.2)	113(8.9)	53(9.9)
Expired	1(0.1)	1(0.1)	0(0)
MBD	1119(86.4)	1097(86.7)	458(85.8)
Transfer	28(2.2)	28(2.2)	16(3.0)
**Triage**			
1	8(0.6)	8(0.6)	2(0.4)
2	33(2.6)	33(2.6)	10(1.9)
3	1065(82.2)	1036(81.8)	454(85.0)
4	189(14.6)	189(14.9)	68(12.7)
**Underlying diseases**			
DM	88(6.8)	88(7.0)	39(7.3)
COPD	8(0.6)	6(0.5)	3(0. 6)
Malignant neoplasms	39(3.0)	40(3.2)	18(3.4)
CKD	11(0.9)	11(0.9)	5(0.9)
Chronic liver disease and cirrhosis	4(0.3)	4(0.3)	2(0.4)

AAD: against-advice discharge; CKD: chronic kidney diseases; COPD: chronic obstructive pulmonary disease; DM: diabetes mellitus; MBD: may be discharge; NA: not available; SD: standard deviation.

Abnormal vital signs, such as high body temperature and fast pulse rate, were observed in most of the test positive patients. The mean arterial pressure (99 mmHg), derived from a patient’s systolic blood pressure (SBP) and diastolic blood pressure (DBP), was within normal range for patients based on the NS1, IgM/IgG, RT-PCR results. Only four (0.31%) of the NS1 test positive patients had relative low blood pressure (SBP < 90 mmHg). At the triage level, most of the patients did not need emergency treatment (triage 1 and 2: 3.2%, triage 3 and 4: 96.3%). There were only 66 patients (4.1%) did not report any medical illness. The majority of patients have other underlying medical deconditions. The average length of stay for ED patients was calculated from the total number of hours spent in ED by patients divided by the total number of discharges, including against-advice discharge (AAD), admission, expired, may be discharged (MBD), and transferred. For patients with NS1 results, the mean duration of stay in the ED was 7.55 hours; the most frequent deposition was direct discharge (86.4%), i.e., MBD, and the average length of stay for MBD patients was 5.61 hours.

### Dengue Fever and Laboratory Results

**Fig 1:**
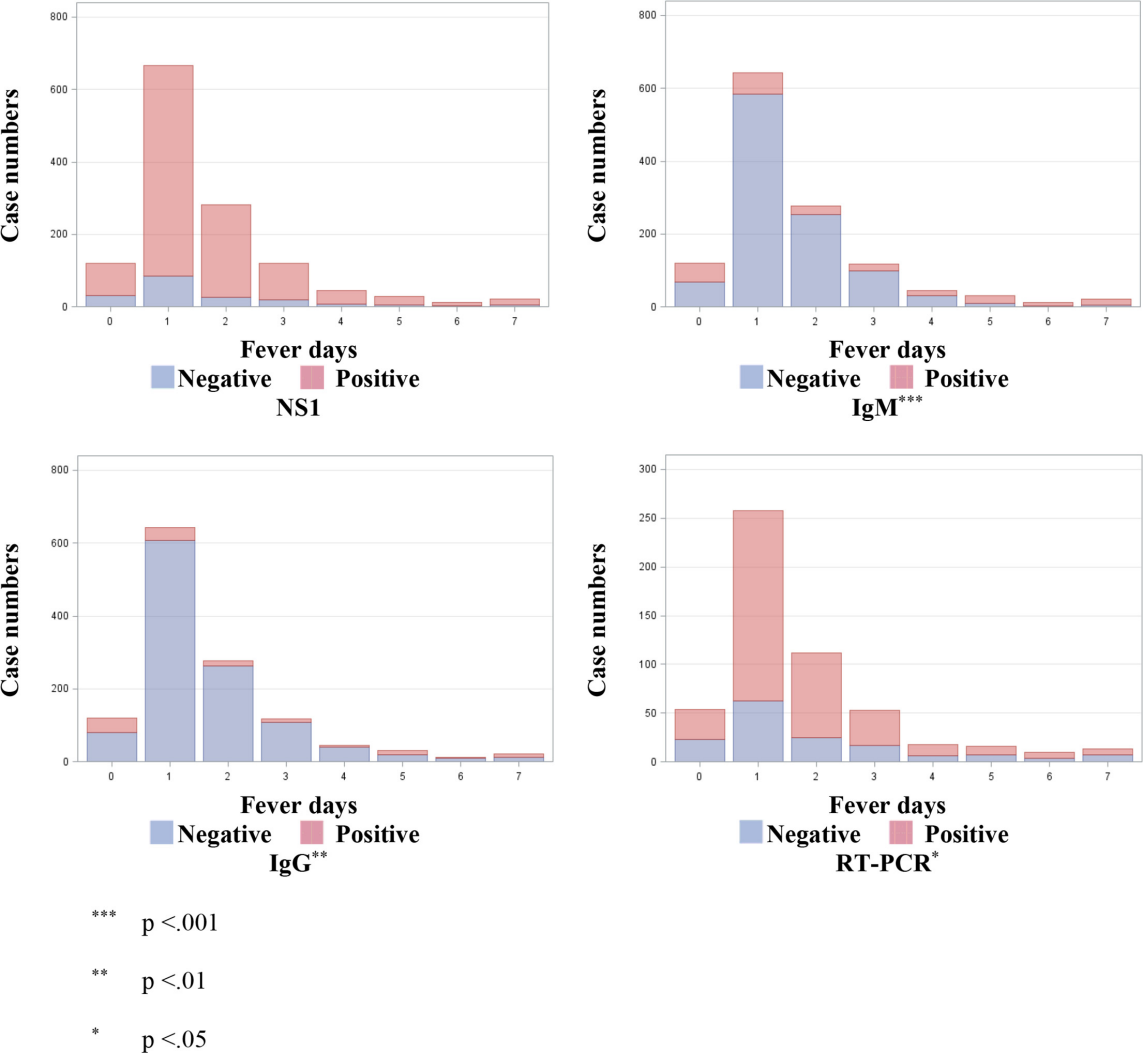
Case numbers of dengue frequency with negative and positive laboratory tests corresponding to fever days examined by Cochran-Armitage trend test. **1A.** NS1. **1B.** IgM. **1C.** IgG. **1D.** RT-PCR. *** p <0.001; ** p <0.01; * p <0.05

The relation between fever days and laboratory test results are summarized in Fig 1. The time-series trends between fever days and positive cases for our gold standard RT-PCR (Fig 1D) reached statistical significance; similar trends were also observed in IgM (Fig 1B), and IgG (Fig 1C); however, the trend from NS1 (Fig 1A) was not significant.

**Fig 2:**
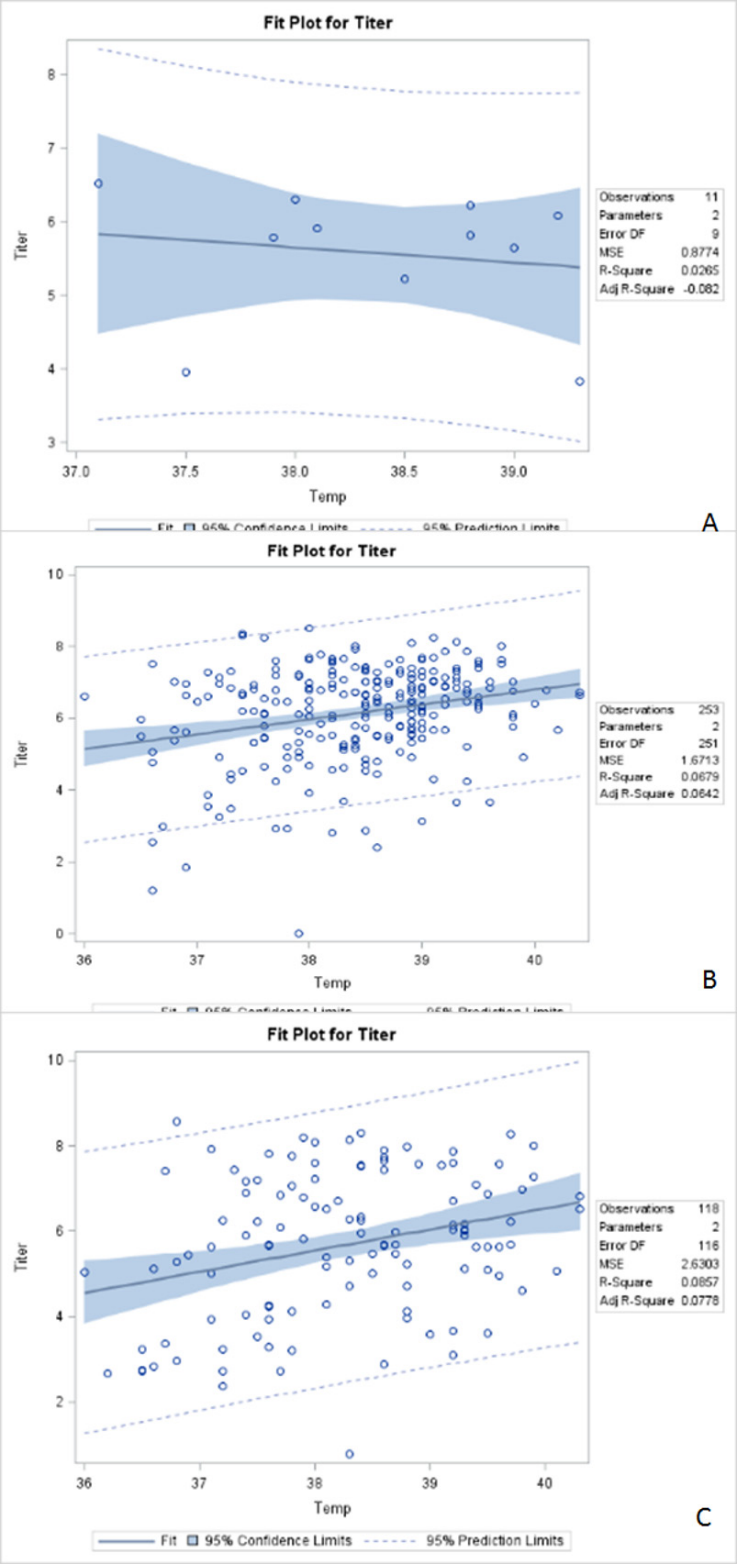
Scatterplots of log-transformed viremia titer by body temperature (Temp) overlaid with the fit line, a 95% confidence band and lower and upper 95% prediction limits. **2A.** 0–17 years. R-Square: 0.0265; Correlation coefficients: -0.30524; p = 0.6327. **2B.** 18–64 years. R-Square: 0.0679; Correlation coefficients: 0.26053; p < 0.0001. **2C.** 65 years & over. R-Square: 0.0857; Correlation coefficients: 0.29269; p = 0.0013

Fig 2 presents the log-transformed viremia titer and body temperature for children (0–17 years), adults (18–64 years), and the elderly (equal to and more than 65 years) depicted as a scatterplot with regression and fitness lines. The R-Square, analyzing how differences in viremia titer can be explained by a difference in body temperature, indicated a small portion of the variation in the slope factor. An additional analysis was applied to evaluate the correlation between the log-transformed viremia titer and body temperature. The correlation analysis indicated a positive association between a viremia titer of dengue and body temperature in the adult (Fig 2B) and elderly (Fig 2C) groups, but no association found in children (Fig 2A) less than 18 years of age. The correlation coefficients were -0.30524 (p = 0.3614) for children, 0.26053 for adults (p <0.0001), and 0.29269 (p = 0.0013) for the elderly.

### Performance of Different Laboratory Strategies

Table 2 shows the examination of the sensitivity, specificity, positive predictive value (PPV), and negative predictive value (NPV) performance for dengue diagnosis and RT-PCR-based composite reference standards. These results for sensitivity, specificity, PPV, and NPV suggested that NS1-based tests have good diagnostic utility for dengue diagnosis (sensitivity: 80.6–100%; specificity: 14–84%; PPV: 60–95%; and NPV: 70–100%). A combination of NS1 test and IgM/IgG test was an applicable dengue screening tool for febrile patients (sensitivity: 95–100%; specificity: 50–68%; PPV: 56–90%; NPV: 86–100%) in the dengue endemic area during the epidemic, which achieved the optimal efficiency for clinical screening in early dengue infection.

**Table 2:** The sensitivity, specificity, positive predictive value (PPV), and negative predictive value (NPV) performance compared with RT-PCR (reference) in acute Dengue infection during 0 to 7 fever days.

	Fever days					
	0	1	2	3	4	5
**NS1**						
Pos./all No.	25/31	187/195	83/87	35/36	12/12	9/9
Sensitivity	80.65	95.90	95.40	97.22	100	100
(95% CI)	(62.53–92.55)^a^	(93.11–98.68)^b^	(91.00–99.80)^b^	(91.85–100.00)^b^		
Pos./all No.	14/23	53/63	19/25	10/17	3/6	1/7
Specificity	60.87	84.13	76	58.82	50	14.29
(95% CI)	(38.54–80.29)^a^	(75.10–93.15)^b^	(54.87–90.64)^a^	(32.92–81.56)^a^	(11.81–88.19)^a^	(0.36–57.87)^a^
Pos./all No.	25/34	187/197	83/89	35/42	12/15	9/15
PPV	73.53	94.92	93.26	83.33	80	60
(95% CI)	(55.64–87.12)^a^	(91.86–97.99)^b^	(88.05–98.47)^b^	(72.06–94.60)^a^	(51.91–95.67)^a^	(32.29–83.66)^a^
Pos./all No.	14/20	53/61	19/23	10/11	3/3	1/1
NPV	70.00	86.89	82.61	90.91	100	100
(95% CI)	(49.92–90.08)^a^	(78.41–95.36)^b^	(61.22–95.05)^a^	(58.72–99.77)^a^		
**IgM**						
Pos./all No.	11/31	9/195	2/87	3/36	4/12	8/9
Sensitivity	35.48	4.62	2.30	8.33	33.33	88.89
(95% CI)	(19.23–54.63)^a^	(1.67–7.56)^b^	(0.00–5.45)^b^	(0.00–17.36)^b^	(9.92–65.11)^a^	(51.75–99.72)^a^
Pos./all No.	16/23	48/63	16/25	9/17	3/6	1/7
Specificity	69.57	76.19	64	52.94	50	14.29
(95% CI)	(47.08–86.79)^a^	(63.79–86.02)^a^	(42.52–82.03)^a^	(27.81–77.02)^a^	(11.81–88.19)^a^	(0.36–57.87)^a^
Pos./all No.	11/18	9/24	2/11	3/11	4/7	9/15
PPV	61.11	37.50	18.18	27.27	57.14	60
(95% CI)	(35.75–82.70)^a^	(18.80–59.41)^a^	(2.28–51.78)^a^	(6.02–60.97)^a^	(18.41–90.10)^a^	(32.29–83.66)^a^
Pos./all No.	16/36	48/234	16/101	9/42	3/11	1/2
NPV	44.44	20.51	15.84	21.43	27.27	50
(95% CI)	(27.94–61.90)^a^	(15.34–25.69)^b^	(8.72–22.96)^b^	(10.30–36.81)^a^	(6.02–60.97)^a^	(1.26–98.74)^a^
**NS1&IgM**						
Pos./all No.	28/31	187/195	83/87	35/36	12/12	9/9
Sensitivity	90.32	95.90	95.40	97.22	100	100
(95% CI)	(62.53–92.55)^a^	(93.11–98.68)^b^	(91.00–99.80)^b^	(91.85–100.00)^b^		
Pos./all No.	13/23	47/63	15/25	8/17	3/6	1/7
Specificity	56.52	74.60	60	47.06	50	14.29
(95% CI)	(34.49–76.81)^a^	(62.06–84.73)^b^	(38.67–78.87)^a^	(22.98–72.19)^a^	(11.81–88.19)^a^	(0.36–57.87)^a^
Pos./all No.	28/38	187/197	83/93	35/44	12/15	9/15
PPV	73.68	94.92	89.25	79.55	80	60
(95% CI)	(59.68–87.68)^a^	(91.86–97.99)^b^	(82.95–95.54)^b^	(64.70–90.20)^a^	(51.91–95.67)^a^	(32.29–83.66)^a^
Pos./all No.	13/16	47/55	15/19	8/9	3/3	1/1
NPV	81.25	85.45	78.95	88.89	100	100
(95% CI)	(54.35–95.95)^a^	(76.14–94.77)^b^	(54.43–93.95)^a^	(51.75–99.72)^a^		
**NS1&IgM&IgG**						
Pos./all No.	28/31	188/195	83/87	35/36	12/12	9/9
Sensitivity	90.32	96.41	95.40	97.22	100	100
(95% CI)	(62.53–92.55)^a^	(93.80–99.02)^b^	(91.00–99.80)^b^	(91.85–100.00)^b^		
Pos./all No.	13/23	43/63	14/25	8/17	3/6	0/7
Specificity	56.52	68.25	56	47.06	50	
(95% CI)	(34.49–76.81)^a^	(55.31–79.42)^a^	(34.93–75.60)^a^	(22.98–72.19)^a^	(11.81–88.19)^a^	
Pos./all No.	28/38	188/208	83/94	35/44	12/15	9/16
PPV	73.68	90.38	88.30	79.55	80	56.25
(95% CI)	(59.68–87.68)^a^	(86.38–94.39)^b^	(81.80–94.80)^b^	(64.70–90.20)^a^	(51.91–95.67)^a^	(29.88–80.25)^a^
Pos./all No.	13/16	43/50	14/18	8/9	3/3	0/0
NPV	81.25	86	77.78	88.89	100	
(95% CI)	(54.35–95.95)^a^	(76.38–95.62)^b^	(52.36–93.59)^a^	(51.75–99.72)^a^		

^a^ Exact confidence limits, in contrast, are based on the binomial distribution, and they have better converage in small samples and/or rare events.

^b^ The confidence limits appearing under the asymptotic standard error (ASE) are based on asymptotic (Wald) theory. If *n* is large, then the 95% confidence interval can be calculated using *p* ± 1.96×*ASE*.

## Discussion

Dengue was classified by the World Health Organization (WHO) as the “most important mosquito-borne viral disease in the world” [15] due to significant geographic spread of the virus and its vector into previously unaffected areas. The WHO guidelines suggested that activities (triage, management decisions, and clinical services) at the primary and secondary care levels (where patients are first observed and evaluated) are critical in determining the clinical outcomes of dengue. A well-managed emergency preparedness and front-line response not only reduce the number of unnecessary hospital admissions but also save the lives of dengue patients. Early notification of dengue cases observed in primary and secondary care is crucial for identifying outbreaks and initiating an early response by applying differential and efficient diagnosis tools [16].

The NS1 test has been proven as a valuable test in increasing the sensitivity of the acute-phase and early-convalescent-phase of dengue diagnosis, surveillance and clinical diagnosis [16–18]. Previous studies suggested the sensitivity and specificity of NS1 Ag were 30–94% and 92–100%, respectively [17–20]. The NS1 Ag assay combined with IgM antibody capture enzyme-linked immunosorbent assay (ELISA) could significantly increase the sensitivity for dengue diagnosis [16]. In asymptomatic individuals, the NS1 Ag capture sensitivity tends to be lower than that in symptomatic patients [17]. A rapid test with Dengue NS1 Ag STRIP (Bio-Rad Laboratories, Marnes-la-Coquette, France) has been applied before in the major Taiwanese health facilities [21] and at airports as on-site detection of imported dengue cases [22]. The use of the Dengue NS1 Ag STRIP was suggested because the diagnosis was made in 11% of patients without the need for second convalescent samples, and 4.3% more cases were detected compared to PCR/ELISA [21]. The data from the airports implied that the NS1 strip rapid test offers several advantages over current routine assays (ELISA and RT-PCR), such as rapidity, simplicity, high sensitivity with a longer detection time for primary infection, and excellent specificity (100%). When applying a first-line test in the diagnosis of dengue in hospitals and at airports, future improvement for the sensitivity used to detect low levels of NS1 antigens in serum would increase the reliability of NS1 strip rapid test, especially for individuals with secondary infections and various dengue virus strains of all four serotypes circulating in different geographic areas. These results also suggested that if the manufacturer can provide antibodies with increased sensitivity for the detection of all four dengue virus NS1 antigens, it would improve the diagnostic value by differentiating primary dengue infection from secondary infection [22]. As results from previous studies, the NS1 Ag, IgM, IgG combined rapid test applied in our study implied the information of previous infection if isolated positive IgG test results were observed and suggested additional benefits of differentiation of primary, secondary type 2 dengue virus infections in endemic areas with increased sensitivity and PPV.

The rapid test would identify patients with atypical dengue presentations. Fever was one of the important reference for patients to access the health care systems. Our study suggested that if fever was used as the indicator of dengue, NS1Ag, alone or either combining IgM, IgG or both would increase the sensitivity and NPV up to 85–100% in the first 5 days. For afebrile cases with additional dengue clinical manifestations, such as malaise and periorbital pain, the sensitivity and NPV of NS1 Ag were also up to 70–81%. Although the specificity of the NS1, either combined with IgM, IgG or both was approximately 70–80% in the first 3 fever days, applying NS1, alone with the IgG, IgM or both as the rapid diagnostic tool was still viable during the dengue epidemic because the acceptable NPV. Furthermore, further blood sampling for dengue confirmation may not necessarily be applied due to the high capability of early detection and a relative long positive period (1–9 days) of the NS1 Ag, IgG/IgM combined rapid test [14]. The long positive period covered the late seroconversion response of IgM and the combined antibody (IgG/IgM) test provide the information of serology prevalence, primary and secondary infections during the epidemic of the endemic area.

Although fever was regarded as one of the important indicators of dengue in an endemic area, dengue should be considered present and screened in afebrile patients, especially in the elderly population. Previous studies suggested fever and myalgia were the most common dengue presentations in the elderly population, and the elderly population might have atypical presentations [23]. Compared to the younger population, the mortality and incidence of severe dengue syndrome were higher in the elderly. Our study suggested the mean viral load of the elderly was high, but the correlation between temperature and viral load was not high. Considering the importance of early diagnosis and treatment in the elderly population to reduce morbidity and mortality, aggressive dengue diagnostic strategies should be considered. Therefore, this highly sensitive rapid diagnosis kit with NS1 alone or combined with IgM and IgG should be widely used as a screening tool for the elderly population, even in asymptomatic patients.

There were several limitations in our study. First, the findings were based on a small number of cases and a single-center teaching hospital center. These two factors resulted in limited precision and limited generalization to infected populations. Second, the study hospital was located in an endemic city with a relatively low dengue prevalence and incidence rate before this epidemic. The predominant dengue virus was DENV type 2. If the study results were extended to other types of dengue virus, more samples need to be obtained from different dengue virus patients. Third, this cross-sectional study neither established a temporal relationship nor distinguished different phases of dengue at different time points in evolving febrile illness [24]. Despite these limitations, our study had a relatively large number of dengue PCR positive patients, and our study highlighted the clinical utility of combining screening tests in describing fever manifestations of dengue that may be crucial and significant.

## Conclusions

Applying the dengue NS1, IgM, and IgG rapid test kit as a diagnostic tool was cost-efficient and a timesaver during the dengue epidemic. Compared with RT-PCR or IgM, IgG ELISA tests that need more laboratory supports, the dengue NS1, IgM, and IgG rapid test kit was much easier to use in the clinical virological laboratory, demonstrating satisfactory sensitivity and cost-effectiveness. Many early actions such as proper treatment in the health care facilities and optimal infection control measurements such as insecticide spray or environmental cleaning in the community could be enacted to achieve the maximal beneficial effects.

## Supporting Information

S1 Raw Data Contains the complete data set. (XLSX)
